# Supplementary material for: Radiation-Induced Innate Neutrophil Response in Tumor Is Mediated by the CXCLs/CXCR2 Axis
Source: Cancers (Basel). 2023 Dec 1;15(23):5686. doi: 10.3390/cancers15235686 (PMC10705172; doi:10.3390/cancers15235686)
Supplement: Supplementary file 1 [file cancers-15-05686-s001.zip › Supplementary Materials S1_Figures.pdf]

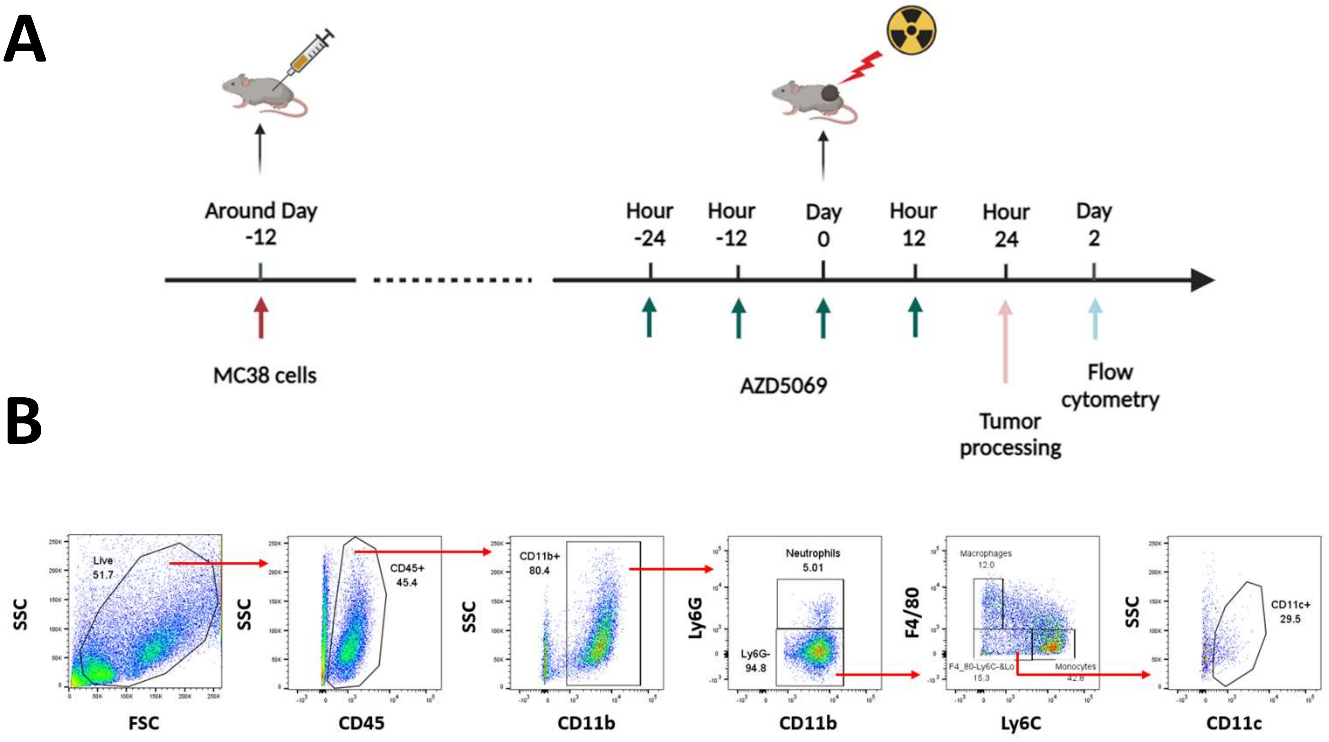

Figure S1. CXCR2 blockade experimental design and flow cytometry gating strategy for the MC-38 tumor model. (A) C57BL/6J mice were injected s.c. with MC-38 tumor cells in the right hind leg. When tumors reached about 7 mm in diameter, mice were randomly assigned to one of four treatment groups and administered with AZD-5069 by oral gavage twice per day for two days. One day after AZD-5069 treatment initiation, tumors were irradiated with a single dose of 15 Gy. Mice were euthanized at 24 hr after irradiation and tumors were collected for flow cytometry analysis. (B) Gating strategy for tumor infiltrating myeloid cell populations in flow cytometry analysis.

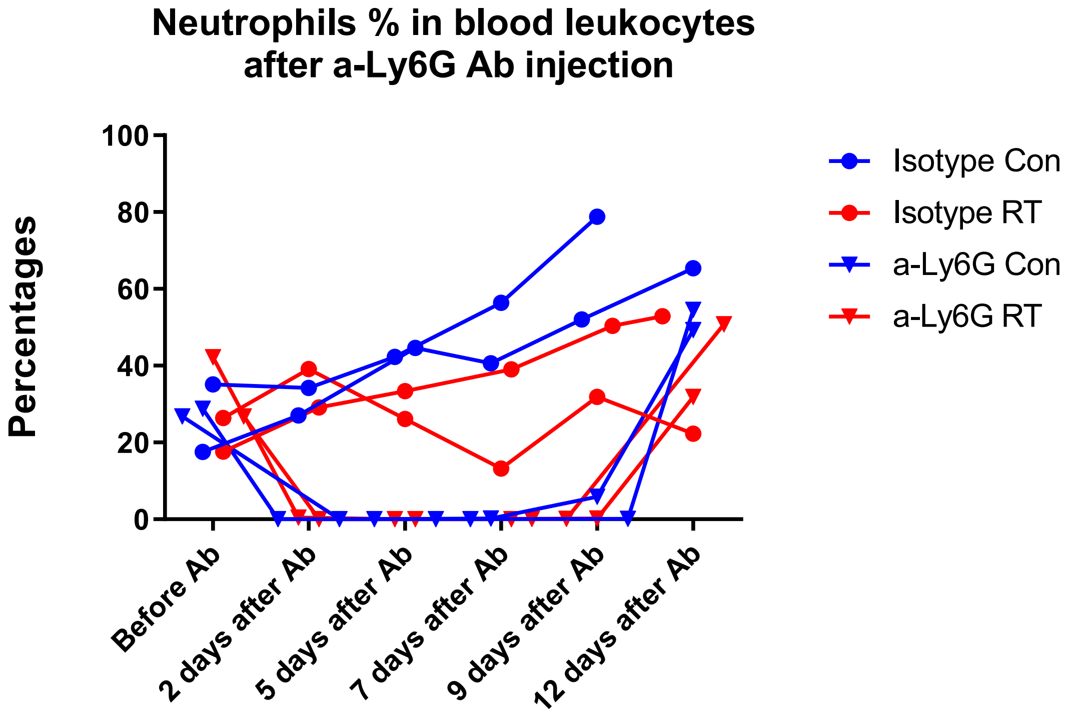

Figure S2. Neutrophil percentages in blood leukocytes at different days after α-Ly6G antibody injection (A) C57BL/6J mice were injected s.c. with RM-9 tumor cells in the right hind leg. When tumors reached about 7 mm in diameter, mice were randomly assigned to four groups and injected intraperitoneally with either α-Ly6G antibody or isotype antibody. Blood samples were collected from tail veins from 2 mice of each group (n=9 or 10) every 2 to 3 days, and flow cytometry was used to analyze the neutrophil levels in the samples.

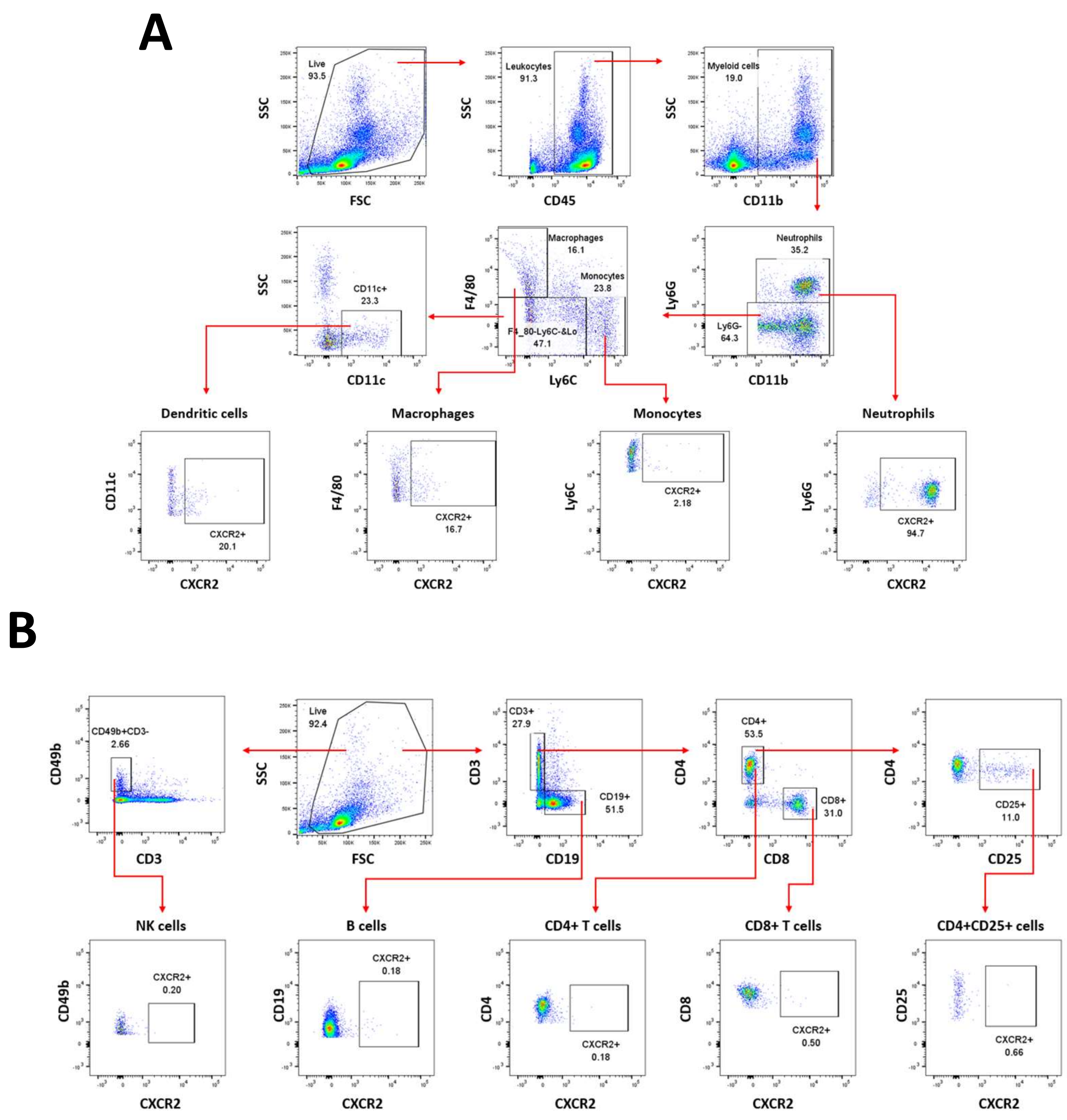

Figure S3. Flow cytometry gating strategies for CXCR2 expression on myeloid and lymphoid populations in the spleen. Spleens from C57BL/6J control and RM-9 tumor-bearing (tumor diameter is about 10 mm) mice were collected and processed for flow cytometry analysis. (A) The gating strategy for myeloid populations. (B) The gating strategy for lymphoid populations. The results for CXCR2 expression are shown in **Figure 3 A-D**.

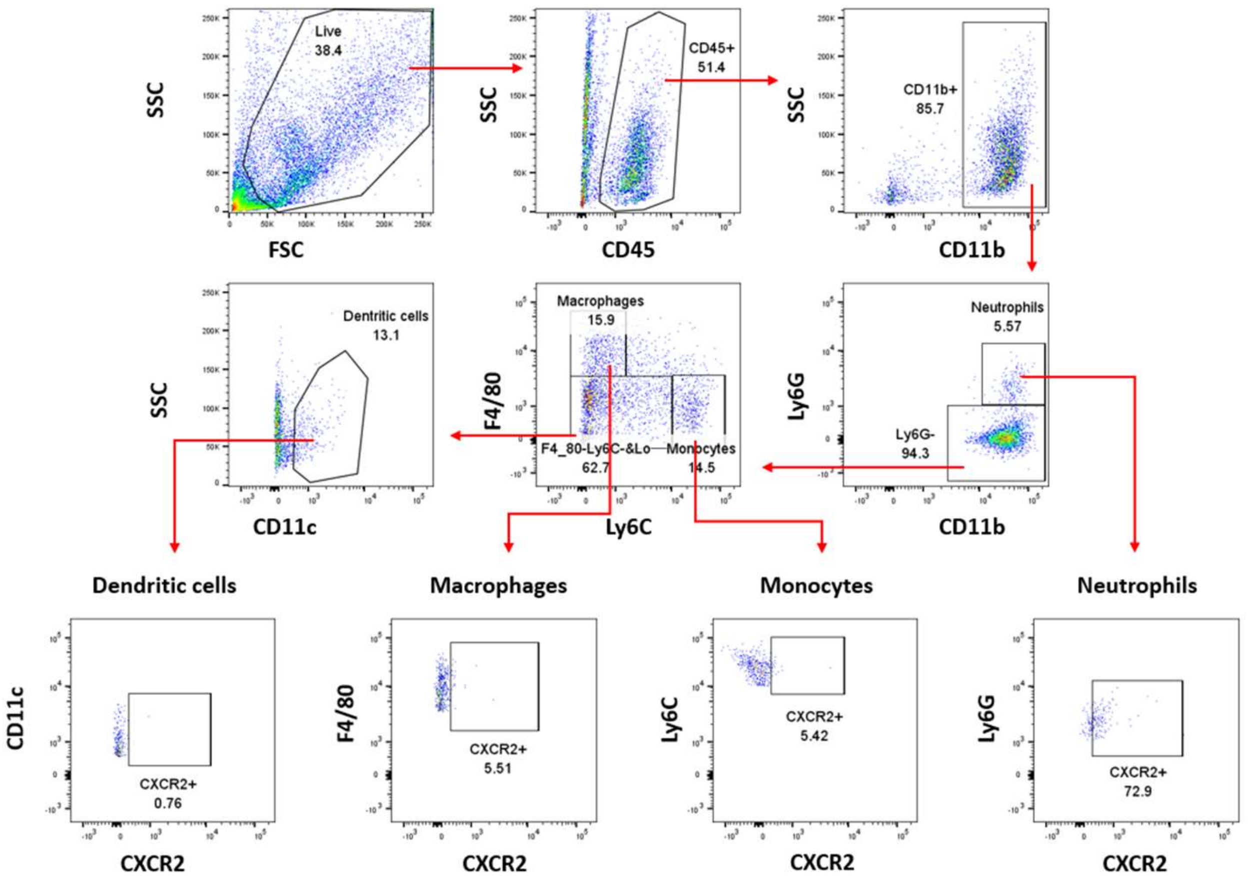

Figure S4. Flow cytometry gating strategies for CXCR2 expression on tumor-infiltrating myeloid populations. Tumors from C57BL/6J control and RM-9 tumor-bearing (tumor diameter is about 10 mm) mice were collected and processed for flow cytometry analysis. The results for CXCR2 expression are shown in **Figure 3 E-F**.

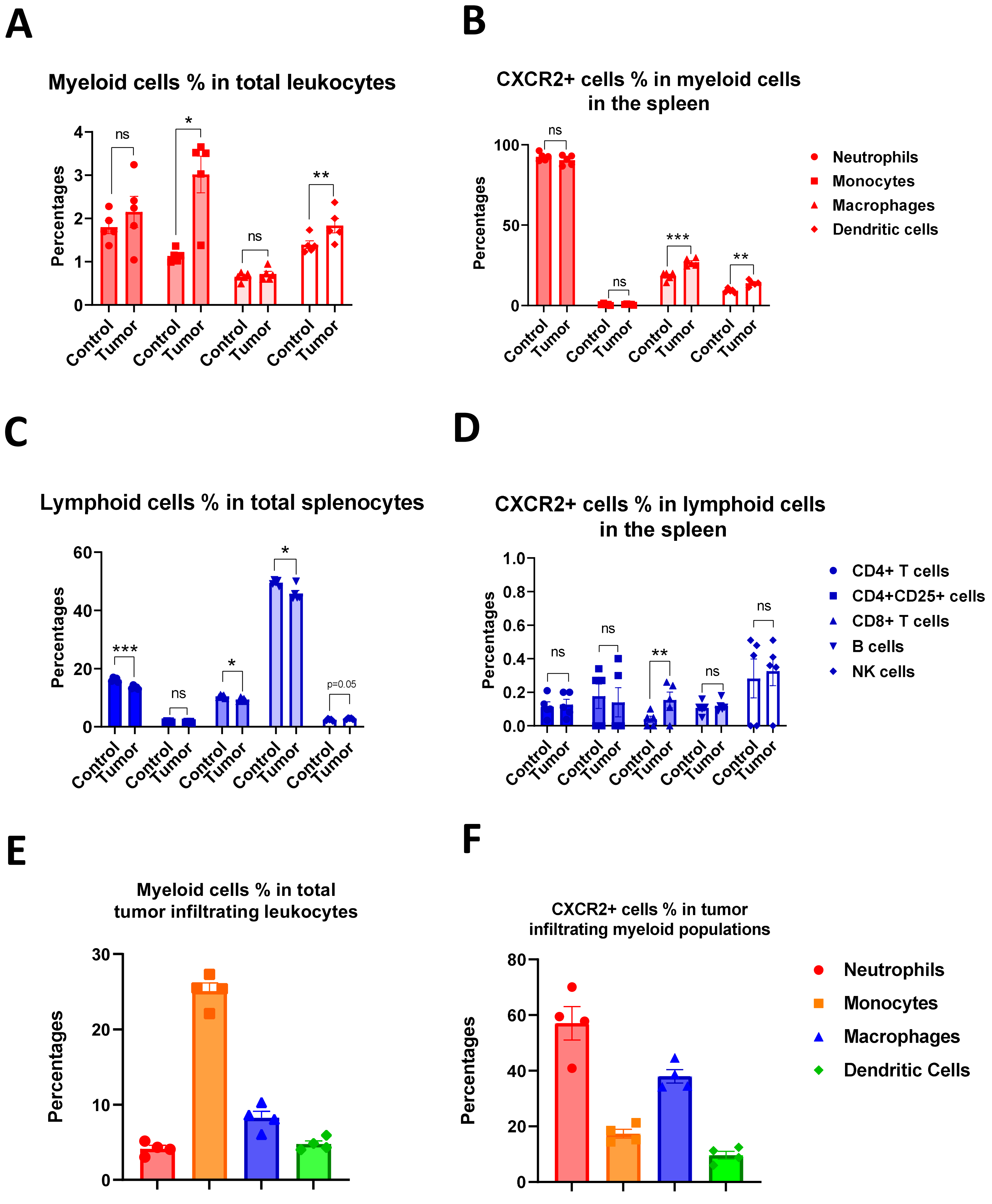
A
Myeloid cells % in total leukocytes
Percentages
0
1
2
3
4
ns
*
ns
**
Control
Tumor
B
CXCR2+ cells % in myeloid cells in the spleen
Percentages
0
50
100
ns
ns
***
**
Control
Tumor
Neutrophils
Monocytes
Macrophages
Dendritic cells
C
Lymphoid cells % in total splenocytes
Percentages
0
20
40
60
***
ns
*
*
p=0.05
Control
Tumor
D
CXCR2+ cells % in lymphoid cells in the spleen
Percentages
0.0
0.2
0.4
0.6
0.8
1.0
ns
ns
**
ns
ns
Control
Tumor
CD4+ T cells
CD4+CD25+ cells
CD8+ T cells
B cells
NK cells
E
Myeloid cells % in total tumor infiltrating leukocytes
Percentages
0
10
20
30
F
CXCR2+ cells % in tumor infiltrating myeloid populations
Percentages
0
20
40
60
80
Neutrophils
Monocytes
Macrophages
Dendritic Cells

Figure S5. CXCR2 is highly and preferentially expressed on neutrophils. For graphs (A) to (D), spleens from C57BL/6J control and MC-38 tumor-bearing (tumor diameter is about 7 mm) mice were collected and processed for flow cytometry analysis. Gating strategies for flow cytometry analysis are the same as RM-9 tumor-bearing mice as shown in **Supplementary Figures 3.** n=5 for both C57BL/6J control and n=4 for MC-38 tumor-bearing groups, values = mean ± SEM. Student's *t*-test was used for comparing the data from control mice with those from tumor-bearing mice. * $p<0.05$, ** $p<0.01$, *** $p<0.001$. (A) Bar graphs showing percentages of different myeloid populations accounting for total splenocytes. (B) Bar graphs showing percentages of $CXCR2^+$ cells in different myeloid populations. (C) Bar graphs showing percentages of different lymphoid populations accounting for total splenocytes. (D) Bar graphs showing percentages of $CXCR2^+$ cells in different lymphoid populations. For graphs (E) and (F), MC-38 tumor tissue (diameter is about 7 mm) were collected and processed for flow cytometry analysis, and gating strategies were the same as RM-9 tumor-bearing mice as shown in **Supplementary Figure 4**. n=5, values = mean ± SEM. (E) Bar graphs showing percentages of different myeloid populations accounting for total tumor-infiltrating leukocytes. (F) Bar graphs showing percentages of $CXCR2^+$ cells in different tumor-infiltrating myeloid populations

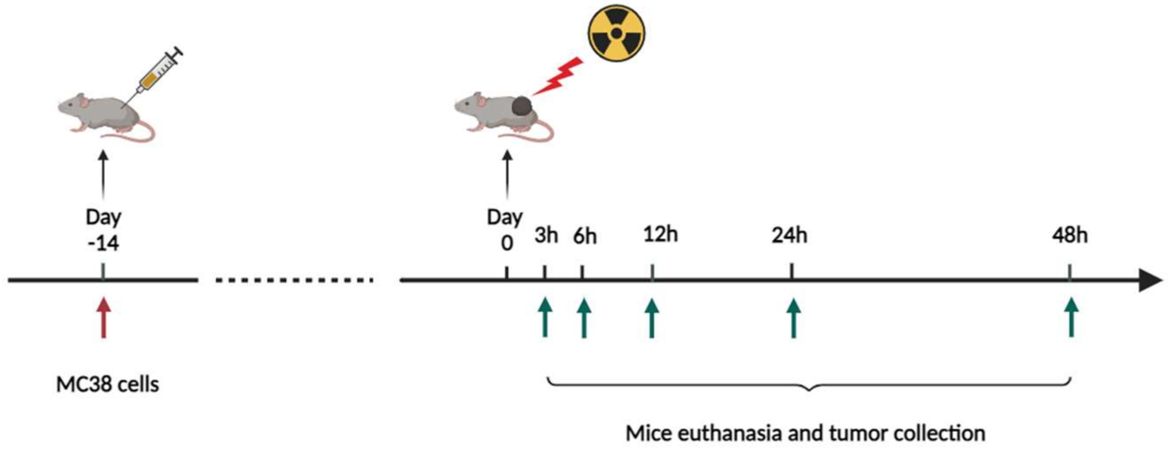

Figure S6. A schematic illustration of the experimental design for MC-38 tumor model. Six groups of mice were injected s.c. with MC38 tumor cells on the right hind leg. When tumor sizes reached about 8 mm in diameter, tumors were irradiated with a single dose of 15 Gy. At 3 h, 6 h, 12 h, 24 h, 48 h post RT, different groups of mice were euthanized, and tumors were collected and stored at -80°C for mRNA sequencing and chemokine multiplex assay.

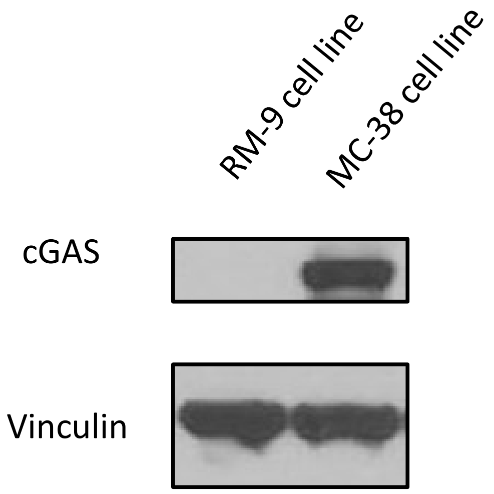

Figure S7. MC-38 tumor cell line expresses cGAS, whereas RM-9 tumor cell line does not express it. RM-9 and MC-38 were cultured in the flasks until reaching 80% confluency. Cells were then harvested for protein extraction and western blot analysis. cGAS expression was examined and vinculin was used as a loading control.

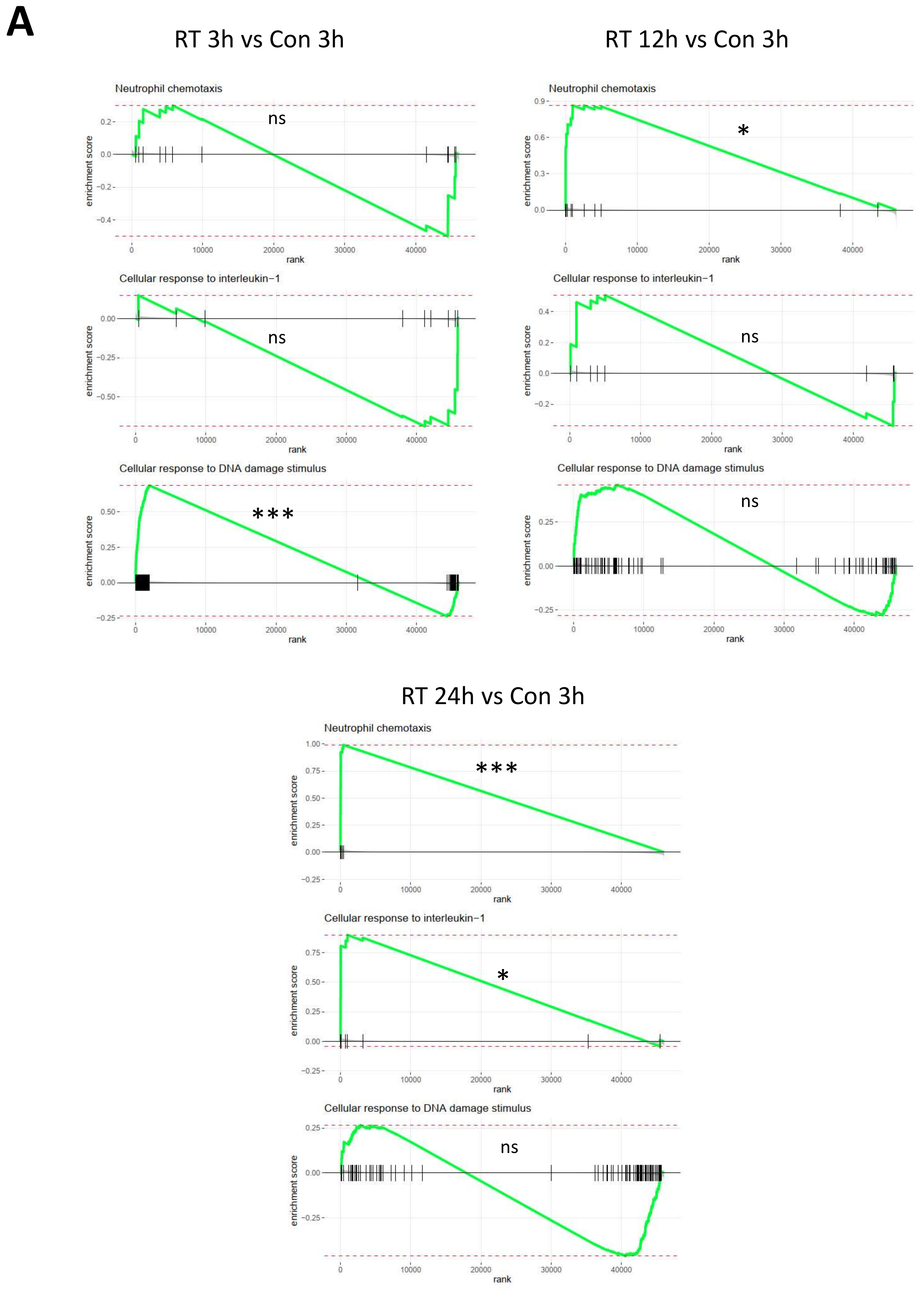
A
RT 3h vs Con 3h
RT 12h vs Con 3h
Neutrophil chemotaxis
ns
*
Cellular response to interleukin−1
ns
ns
Cellular response to DNA damage stimulus
***
ns
RT 24h vs Con 3h
Neutrophil chemotaxis
***
Cellular response to interleukin−1
*
Cellular response to DNA damage stimulus
ns
enrichment score
rank

B

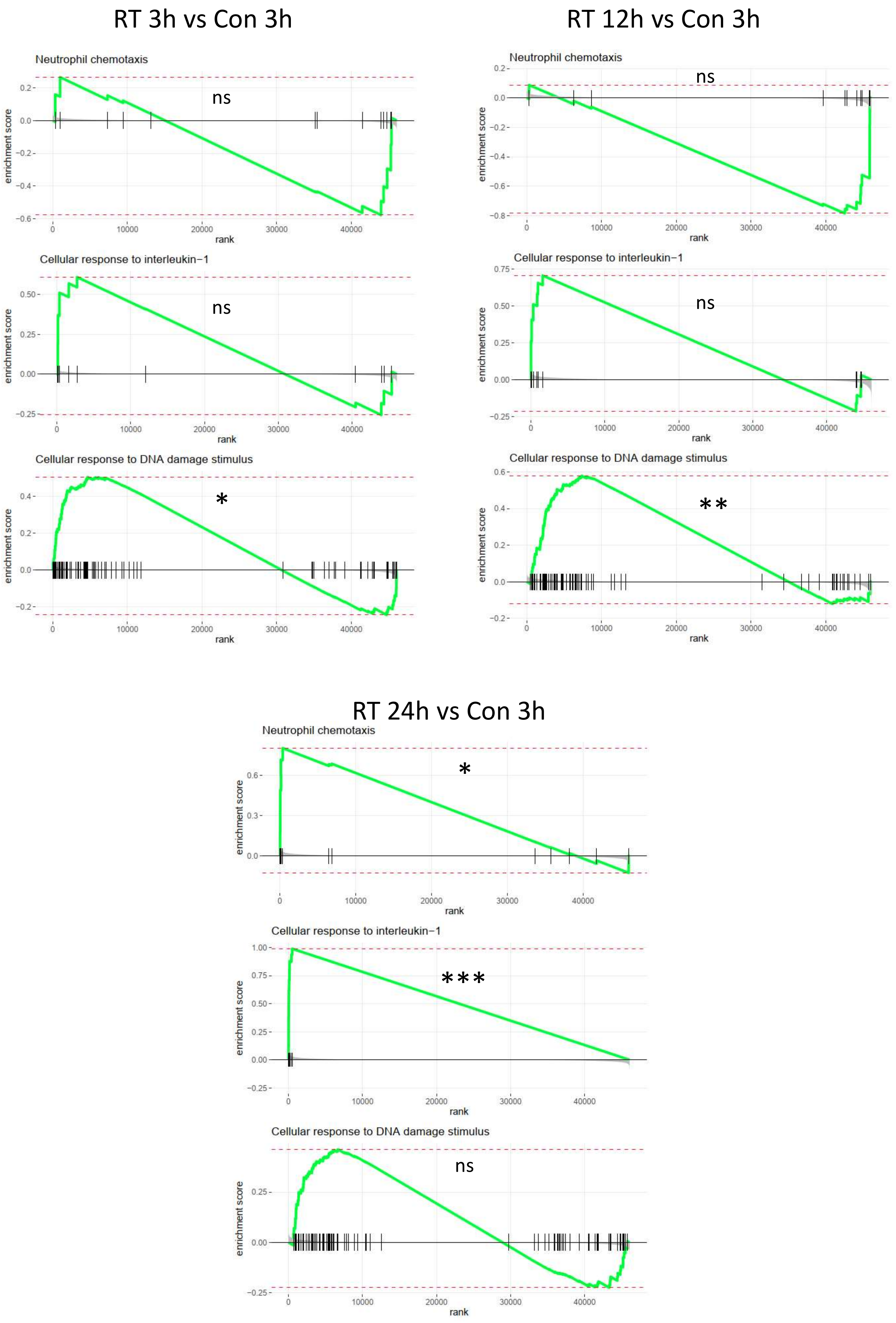
RT 3h vs Con 3h
RT 12h vs Con 3h
RT 24h vs Con 3h
Neutrophil chemotaxis
Cellular response to interleukin−1
Cellular response to DNA damage stimulus
enrichment score
rank
ns
*
**
***

Figure S8. Plots from gene set enrichment analysis. (A) Plots from RM-9 model. (B) Plots from MC-38 model. ns = not significant. * p<0.05, ** p<0.01, *** p<0.001.
