# Supplementary material for: Radiation-Induced Innate Neutrophil Response in Tumor Is Mediated by the CXCLs/CXCR2 Axis
Source: Cancers (Basel). 2023 Dec 1;15(23):5686. doi: 10.3390/cancers15235686 (PMC10705172; doi:10.3390/cancers15235686)
Supplement: Supplementary file 1 [file cancers-15-05686-s001.zip › Supplementary Materials S3_Densitometry readings and radios of western blot bands.pdf]

Densitometry readings and radios of western blot bands for Figure 5E.

Bands from left to right	Vinculin	IL-1β	IL-1β/Vinculin
1	8599.11	2127.99	0.25
2	8313.87	1140.87	0.14
3	8970.11	1667.70	0.19
4	8233.99	2839.58	0.34
5	9304.23	6257.82	0.67
6	6674.82	3038.79	0.46
7	6166.53	1483.06	0.24
8	7872.06	3929.41	0.50
9	5837.70	2143.28	0.37
10	9043.53	8910.06	0.99
11	8815.99	7476.41	0.85
12	5905.41	3670.70	0.62
13	7665.23	2298.70	0.30
14	6268.70	4547.53	0.73

Densitometry readings and radios of western blot bands for Figure 5F.

Bands from left to right	Vinculin	IL-1β	IL-1β/Vinculin
1	6185.16	2503.65	0.40
2	7892.16	2370.96	0.30
3	7025.70	2406.79	0.34
4	7394.41	2057.75	0.28
5	8109.99	3470.99	0.43
6	7076.58	5849.28	0.83
7	7337.11	5532.89	0.75
8	6855.63	9825.94	1.43
9	6573.16	6197.31	0.94
10	8806.41	7144.99	0.81
11	7613.41	3852.23	0.51
12	7102.87	1929.87	0.27
13	8688.99	6662.11	0.77
14	8683.70	7152.11	0.82
15	8862.70	8012.36	0.90
