# Supplementary figures and images for: Radiation-Induced Innate Neutrophil Response in Tumor Is Mediated by the CXCLs/CXCR2 Axis
Source: Cancers (Basel). 2023 Dec 1;15(23):5686. doi: 10.3390/cancers15235686 (PMC10705172; doi:10.3390/cancers15235686)

1. The original blots for Figure 5E.

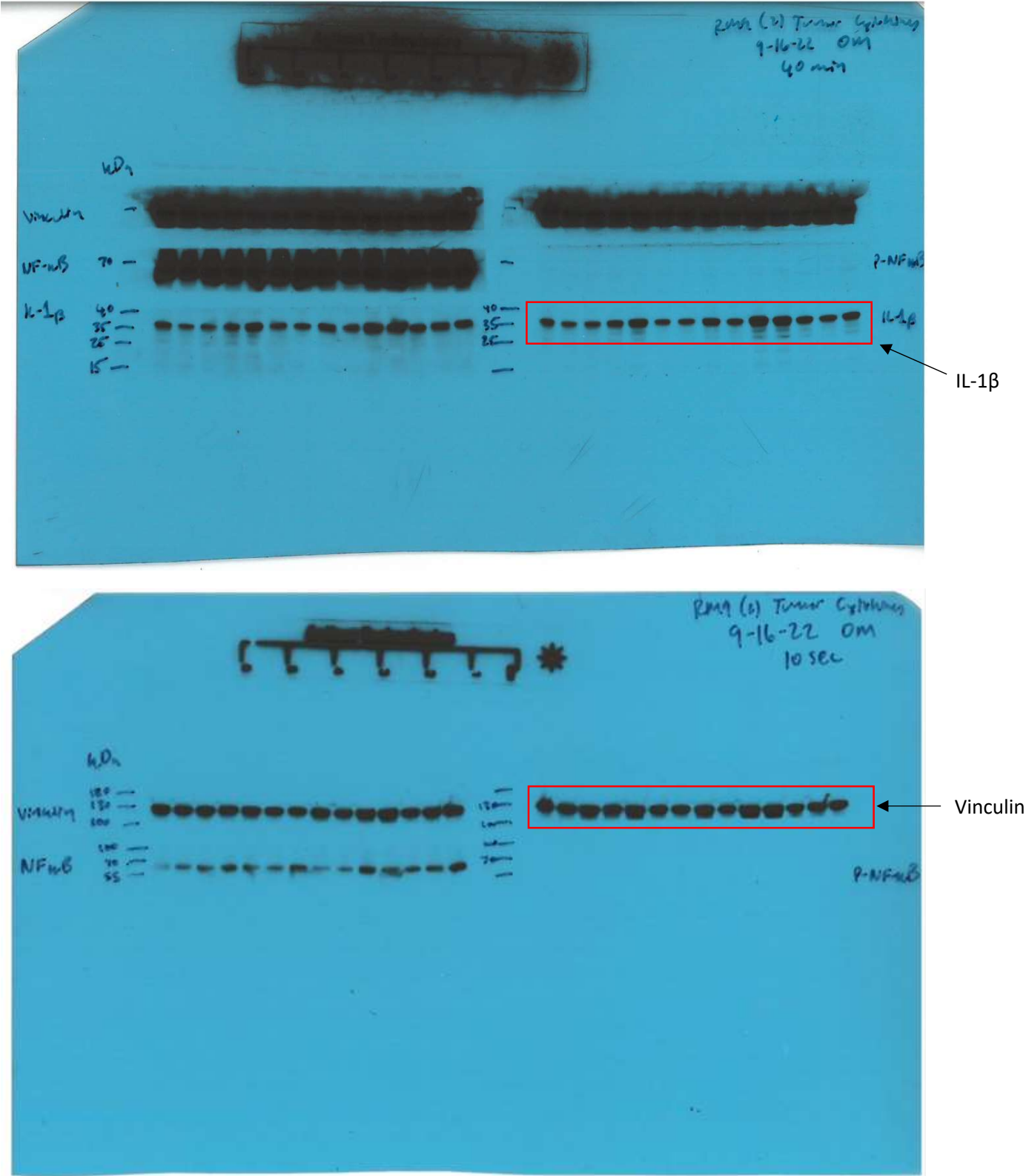

2. The original blots for Figure 5F.

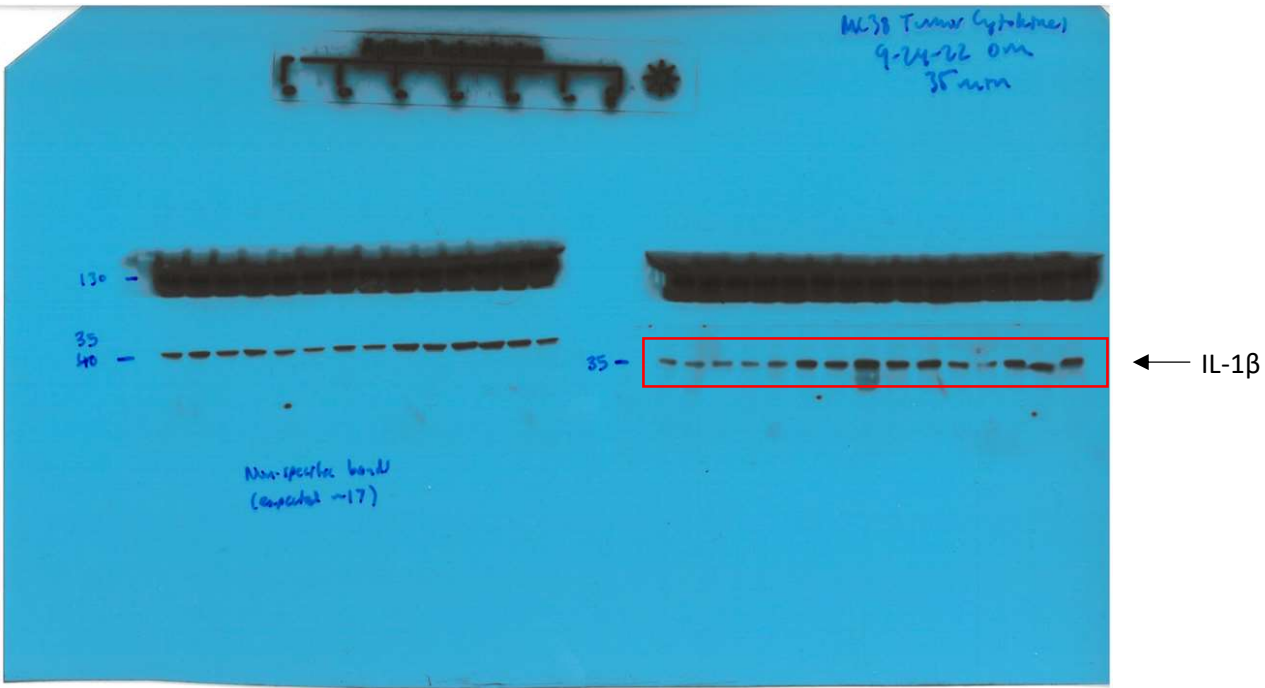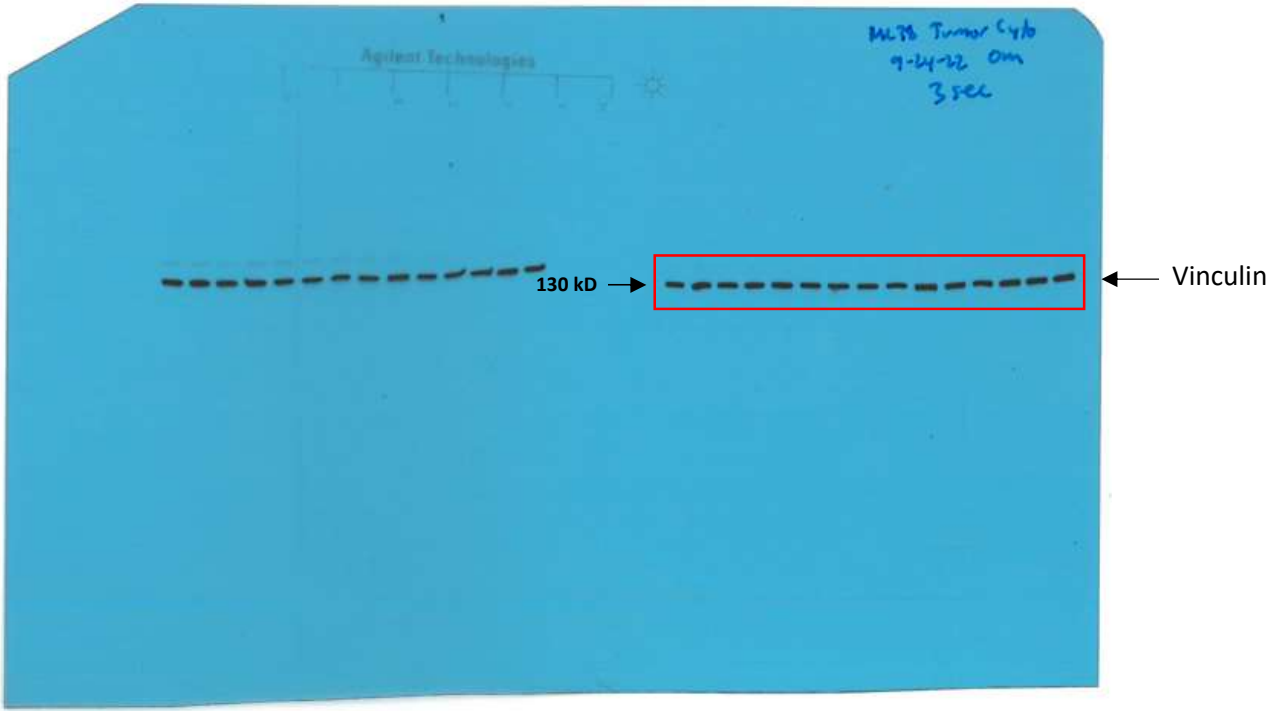

3. The original blots for Figure S7.

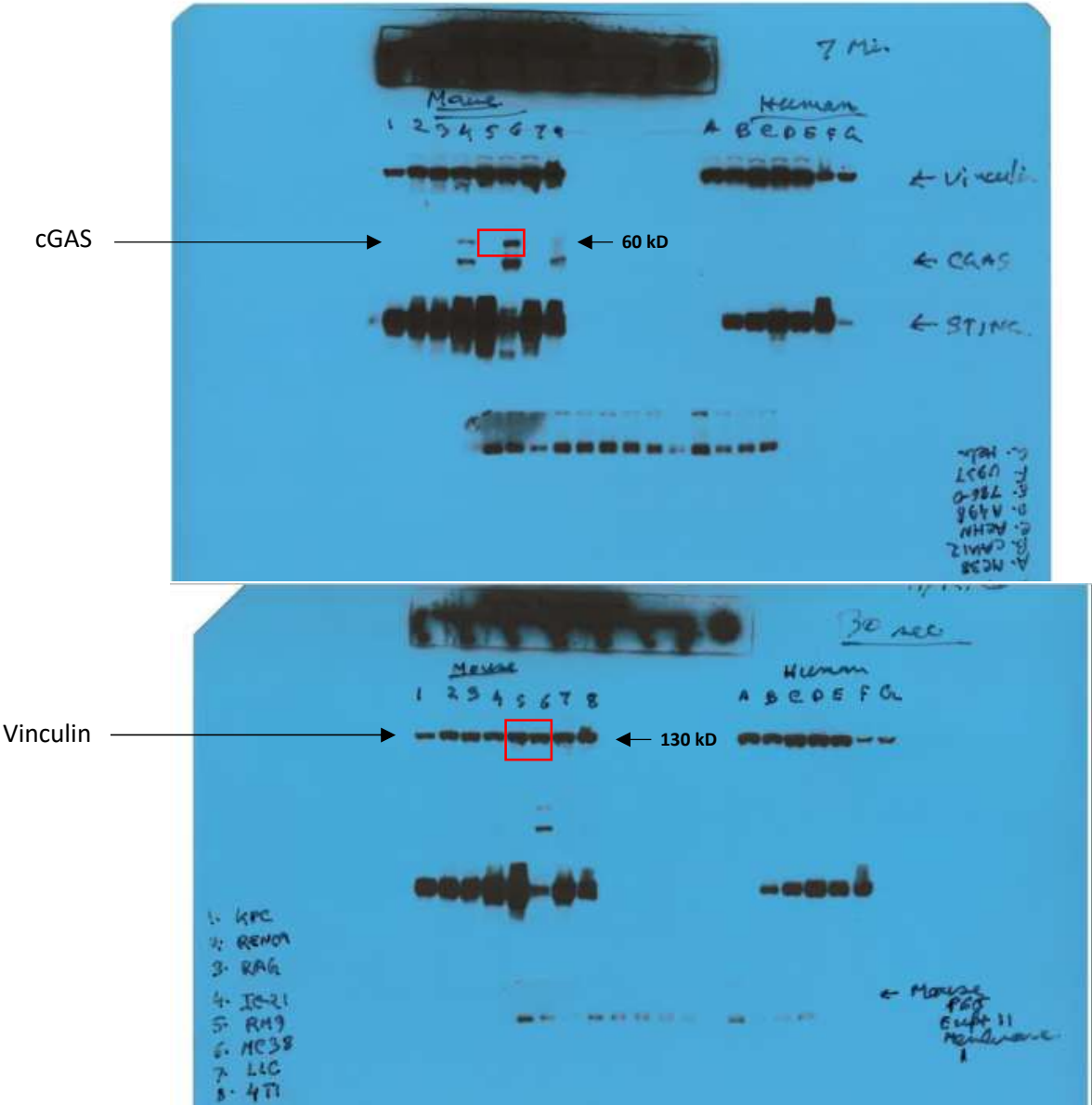

Supplement: Supplementary file 1 [file cancers-15-05686-s001.zip › Supplementary Materials S2_Original western blots_updated.pdf]
